# Progress in Anæsthetics

**Published:** 1903-07-18

**Authors:** 


### Progress in Anæsthetics.

### ANÆSTHESIA.

The rarity of fatalities under nitrous oxide anæsthesia makes it all the more important, not only that every such case be reported, but that every endeavour be made to ascertain whether the cause of such accident lie in the poisonous effect of the anæsthetic itself, the condition of the patient, or the administration of the gas.

In a case reported by Dr. Maughan,[1] sudden death occurred in a young woman to whom was administered nitrous oxide gas in the ordinary way for the simple incision of the tonsil for acute tonsillitis. Respiration ceased, and in spite of everything being done, even laryngo-tracheotomy with artificial respiration, the patient died. Though the breathing was at first restarted, it ceased after 15 minutes.

Mr. Walter Tyrrell, commenting on this case, stated that death was due to a toxic condition of the heart being overstrained by the anæsthetic. Dr. Frederick Hewitt,[2] in his Emeritus lectures, states that to patients with angina ludovici nitrous oxide gas may be a direct poison, and even in normal hearts the strain imposed by a full dose of this gas may sometimes be detrimental. So it would seem that special care must be exercised in the choice of an anæsthetic in these throat cases. That respiration should have been restarted and then should have failed again is similar to a case of death under chloroform anæsthesia instanced by Dr. Low.[3] The patient was only lightly anæsthetised when respiration failed, but was restarted artificially, and the patient recovered sufficiently to answer questions, yet died a few minutes later.

Dr. Bodine,[4] of New York, believes that when it is considered how large a part fright plays in the safety or otherwise of anæsthetics, chloroform is as safe as ether. He quotes two cases of death from fright before the administration of any anæsthetic at all, and points out how many deaths take place at the very outset of the administration before more than a few drops have been given. Eliminate fright and chloroform becomes much safer. Parturient women and children take it well and safely as a rule. He thinks that much more care should be taken to ensure absolute quiet, etc., before the commencement of the administration.

Dr. Hewitt[5] in his Emeritus lectures deals with the subject of the anæsthetisation of difficult and bad subjects. In order to have a basis to start from he describes as a normal or perfect type of case for anæsthetics "the middle-aged woman in moderately good health, of spare build, sallow complexion, placid temperament, and moderate habits, possessing a free nasal air-way, and somewhat defective teeth." It is a common thing to suppose that the stronger the heart-beat the safer the anæsthetisation, but Dr. Hewitt states that in general terms it may be said that the patient with a weak heart may be a better subject than the patient with a strong one. He divides his difficult and bad subjects into six classes :—

1. Patients whose general health is good, possibly exceedingly good, but who possess some physical peculiarity rendering them more or less liable to intercurrent embarrassment, or arrest of breathing.
2. Patients whose respiratory tract is in some way encroached upon, or whose respiration is in some way hampered by pathological or other conditions.
3. Patients suffering from certain grave visceral or constitutional affections.
4. Highly nervous or excitable patients; those suffering from various nervous affections; and those who have become addicted to the excessive use of alcohol, tobacco, morphine, or other drugs.
5. Patients displaying in a state of combination or association the characteristics of two or more of the foregoing classes.
6. Patients who prove to be difficult or bad subjects without any discoverable cause.

In speaking of the fourth class he refers[6] to the great influence that the excessive use of tobacco has in disturbing and modifying the usual phenomena of anæsthesia. "Great smokers," he says, "may be quite as difficult to anæsthetise as great drinkers." Repeated swallowing, hesitating, and suspended breathing, the excessive secretion of mucus, inconvenient muscular rigidity, especially about the jaws and neck, obstructive stertor, dilated pupils and exaggerated reflexes are common incidents in the anæsthetisation of these patients." The toleration of such large doses of the anæsthetic may be erroneously attributed to alcohol.

After the use of Schneiderlein's morphine-scopolamine in 11 operative cases, Schicklberger[7] considers that its use is disappointing in that the narcosis is not deep enough. It might, however, he says, be useful in cases where chloroform is contra-indicated. In 7 out of the 11 cases general inhalation anæsthesia had to be resorted to, and it is of great disadvantage to have to wait four hours before the commencement of the operation, and stronger doses cannot be used owing to the toxic nature of the drug.

Chevalier [8] speaks highly of anæsthesine as a local anæsthetic. It is the ethylic ether of paramidobenzoic acid. An odourless white powder, only slightly soluble in water, but readily soluble in alcohol, ether, chloroform, acetones, fats and oils, and is not decomposed by fats. Oil of sweet almonds dissolves it to 3 per cent. of its own volume.

Comparing it with orthoform he finds that on animals it is less toxic. It is satisfactory as a local anæsthetic, and can be used internally. Thus ·03–·05 gr., twice or thrice daily, can be given for nervous dyspepsia and ulceration of the stomach, and its sedative effect is greater and more lasting than that of orthoform.

Made into a pomade with lanolin it is a useful anti-pruritic in diabetic and senile pruritus. In rectal suppositories it relieves pain without affecting tenesmus. For laryngeal work Von Noorden considers it the most satisfactory local anæsthetic he has used; and Kossel uses it in laryngeal work, giving it in the following form, viz.:—

| | | |
|---|---|---|
| Anæsthesine ... ... | ... parts | 20 |
| Menthol ... ... ... | „ | 10-20 |
| Ol. olivæ ... ... ... | „ | 100 |

1 Brit. Med. Jour., March 7, 1903. 2 Lancet, Jan. 17, 1903. 3 Brit. Med. Jour., March 7. 4 Brit. Med Jour., Feb. 21, 1903. 5 Lancet, Jan. 10, 1903. 6 Lancet, Jan. 17. 7 Brit. Med. Jour., Jan. 23. 8 Brit. Med. Jour., May 7.

